# OA12.04. The role of mind-body awareness in complementary and alternative medicine (CAM) outcomes

**DOI:** 10.1186/1472-6882-12-S1-O48

**Published:** 2012-06-12

**Authors:** F Sirois, C Bann, E Walsh

**Affiliations:** 1 Bishop's University, Sherbrooke, Canada; 2 RTI International, Research Triangle Park, USA

## Purpose

Body awareness - attending to, and identifying the inner sensations and overall state of the body and its changes in response to emotional and environmental shifts - is often viewed as an outcome of CAM use. Emerging evidence suggests that mind-body awareness may be an intermediate outcome that contributes to CAM-related outcomes. Qualitative and quantitative work indicate that provider empowerment and support may facilitate the development of mind-body awareness associated with CAM use. Consistent with a whole systems research perspective, the aim of the current study was to test a model of mind-body awareness as an intermediate outcome of CAM use via provider autonomy support that facilitates improvements in self-reported symptoms and health behavior changes.

## Methods

A sample of 243 undergraduate students (mean age = 23.5, 84% female) screened for current use of CAM completed a survey including questions about their CAM use, perceived health-related outcomes from their use of CAM, a measure of CAM provider autonomy support, and a new 8-item measure of Mind-body Awareness (MBA).

## Results

Bivariate analyses confirmed the associations among MBA, autonomy support, CAM use and positive CAM-related health behavior (diet, weight loss, exercise) and symptom (sleep quality, mood, energy levels, concentration) changes. Path analysis controlling for demographics tested the proposed model of CAM use predicting provider autonomy support, which in turn predicts MBA and the two CAM-related outcomes. The model fit well to the data, CFI = 0.96, TLI = 0.93, RMSEA = .03, supporting the hypotheses that CAM use enhances MBA via increased autonomy support, and MBA contributes to positive symptom and health behavior changes from CAM use.

## Conclusion

Our findings extend previous research on body awareness by linking it to CAM-related symptom and behavioral outcomes in a sample of young adult CAM consumers, and further suggest a route through which provider support may enhance CAM outcomes.

